# 

**Published:** 1904-03

**Authors:** 


THE AMERICAN

### Journal of Dental Science.

THIRD SERIES.

THE OLDEST DENTAL MAGAZINE IN THE WORLD.

VOL. XXXV. MARCH, 1904. NO. 3.

EDITOR:
WM. GIRD BEECROFT, D. D. S., Madison, Wis.
ASSOCIATE EDITORS:
JOHN P. BUCKLEY, Ph. G., D. D. S., Chicago.
ALBERT H. KETCHAM, D. D. S., Denver, Colo.
EMORY A. BRYANT, D. D. S., Washington, D. C.
Published on the 10th day of each month.
Subscription Price, $1.00 per year.
Foreign Countries, $1.50 per year.
Address all communications, both business and editorial to the editor.
**Entered as Second Class Matter, Madison, Wis.**

### BOOK NOTICE.

Compend of Dental Pathology and Dental Medicine. By George W. Warren, A. M., D. D. S. Fourth edition. Illustrated. P. Blakeston's Son & Co., 1012 Walnut street, Philadelphia, Pa. Price, 80 cents.

This compend contains the most noteworthy points upon the subjects of interest to the dental student and practitioner, and a section on emergencies. It is a work in which knowledge of all that pertains to dental medicine and dental pathology is in concentrated form, easy of access to the student in his classes and the busy dentist at his chair. It is a book that should be ever at hand, in the pocket of the one and in the cabinet of the other, for ready reference. A compend may be said to be simply an index to a large work on a scientific subject. It is that and a great deal more, as it gives the gist of the subject matter in reference, and also maps out as it were its complete study. Such is the compend at hand. Concise, yet diffusive. It is well up-to-date, reliable and comprehensive.

